# Modulation of Skin Inflammatory Response by Active Components of Silymarin

**DOI:** 10.3390/molecules24010123

**Published:** 2018-12-30

**Authors:** Jana Juráňová, Juliette Aury-Landas, Karim Boumediene, Catherine Baugé, David Biedermann, Jitka Ulrichová, Jana Franková

**Affiliations:** 1 Department of Medical Chemistry and Biochemistry, Faculty of Medicine and Dentistry, Palacky University, Hněvotínská 3, 775 15 Olomouc, Czech Republic; janajuranova@centrum.cz (J.J.); jitkaulrichova@seznam.cz (J.U.); 2 Institute of Molecular and Translational Medicine, Faculty of Medicine and Dentistry, Palacky University, Hněvotínská 5, 779 00 Olomouc, Czech Republic; 3 EA7451 BioConnecT, Normandie University, UNICAEN, 14000 Caen, France; juliette.aury-landas@unicaen.fr (J.A.-L.); karim.boumediene@unicaen.fr (K.B.); catherine.bauge@unicaen.fr (C.B.); 4 Institute of Microbiology of the Czech Academy of Sciences, Laboratory of Biotransformation, Vídeňská 1083, 14220 Praha 4, Czech Republic; david.biedermann@gmail.com

**Keywords:** fibroblasts, inflammation, skin wound healing, cytokines, NF-κB

## Abstract

In this study, we compared selected silymarin components, such as quercetin (QE), 2,3-dehydrosilybin (DHS) and silybin (SB), with the anti-inflammatory drug indomethacin (IND) in terms of their wound healing potential. In view of the fact that pathological cutaneous wound healing is associated with persistent inflammation, we studied their anti-inflammatory activity against inflammation induced by bacterial lipopolysaccharide (LPS). We investigated the regulation of crucial pro-inflammatory transcription factors—nuclear factor kappa-B (NF-κB) and activator protein 1 (AP-1)—as well as the expression of downstream inflammatory targets by Western blotting, real-time PCR (RT-PCR), electrophoretic mobility shift assay (EMSA), and/or enzyme-linked immunosorbent assay (ELISA) in vitro using primary normal human dermal fibroblasts (NHDF). We demonstrated the greater ability of DHS to modulate the pro-inflammatory cytokines production via the NF-κB and AP-1 signaling pathways when compared to other tested substances. The prolonged exposure of LPS-challenged human dermal fibroblasts to DHS had both beneficial and detrimental consequences. DHS diminished interleukin-6 (IL-6) and interleukin-8 (IL-8) secretion but induced the significant upregulation of IL-8 mRNA associated with NF-κB and AP-1 activation. The observed conflicting results may compromise the main expected benefit, which is the acceleration of the healing of the wound via a diminished inflammation.

## 1. Introduction

Skin wound healing is a highly organized process of tissue regeneration that requires the interplay of skin resident cells, e.g., keratinocytes and fibroblasts, and recruited leukocyte subtypes participating in three sequential but overlapping phases: Inflammation, proliferation, and maturation [1]. Typically, cutaneous wound repair is complicated by bacterial infection, which can lead to the continuous infiltration of neutrophils, thereby delaying the wound from healing or halting the process indefinitely. Therefore, the transition from the inflammatory to proliferative phase is an essential step during wound healing and the optimization of inflammation is targeted by current treatments.

The sensing of microbial infection by transmembrane proteins such as toll-like receptors (TLRs) triggers intracellular pro-inflammatory pathways, including the nuclear factor kappa-light-chain-enhancer of activated B cells (NF-κB), mitogen-activated protein kinases (MAPK) pathways resulting in the expression of pro-inflammatory cytokines e.g., interleukin-1 (IL-1) family of cytokines or interleukin-6 (IL-6), chemokines (IL-8) and enzymes such as cyclooxygenase-2 (COX-2) [2,3,4]. These soluble mediators organize the inflammatory response by attracting neutrophils. Neutrophils integrate the network of cellular interactions in order to maintain an immune homeostasis. In this way, they ensure a seamless transition to subsequent wound healing phases and the final restoration of tissue homeostasis [2]. On the other hand, the dysregulated excessive or persistent production of these inflammatory mediators generates a sustained pro-inflammatory state, resulting in tissue damage [3,4].

A common approach to reduce the inflammation is the utilization of competitive inhibitors of COX, non-selective or COX-2 selective nonsteroidal anti-inflammatory drugs (NSAID) having anti-inflammatory and antipyretic properties [5]. Interestingly, those effects may be mediated independently of COX activity, but via the NSAID attenuation of the activation of NF-κB or the activator protein 1 (AP-1) [6,7]. However, NSAID application has been linked to serious side effects [8,9,10].

Over the past decade, there has been a growing public interest in herbal medicine [11,12]. Silymarin, the standardized extract of the milk thistle fruit (*Silybum marianum* (L.) Gaertn.) and its main component silybinin are generally accepted as very safe [13]. According to the European Union’s herbal monograph on *Silybum marianum* (L.) Gaertn. fructus from 2018, the powdered herbal substance is used in a single dose between 300 mg to 600 mg and the dry extract in a single dose of about 200 mg. Silymarin contains several flavonolignans (in this case taxifolin coupled variously with coniferyl alcohol), namely silybin A, silybin B, silychristin A, isosilybin A and silydianin. The natural diastereomeric mixture of silybin A and silybin B, roughly 1:1, is often denoted as silybinin. This is the name used in pharmacopoeias. Besides its well-known hepatoprotective activity, silymarin has been reported to be a promising anticancer [13,14,15], antidiabetic [16,17,18], cardioprotective [19,20] and neuroprotective [21] agent. Considering the beneficial effects of silymarin when used as a dietary supplement, a topical administration has been examined [22]. The diminished chemically induced contact dermatitis in mice [23] or in vitro bacterial inflammation has been observed following the application of silymarin [24]. Within the last decade, depending on more precise techniques for identification and separation, an enlarging group of active compounds of silymarin—including quercetin (QE) [12,25,26] or dihydroquercetin (also known as taxifolin) [27]—has been proposed to be effective in inflammatory and wound healing processes. However, these flavonoids are not strictly contained only in silymarin.

Flavonolignan SB, the main component in most preparations of silymarin, easily oxidizes to 2,3-dehydrosilybin (DHS) [28,29]. DHS, despite its minor presence in silymarin [30], possesses a higher therapeutic regenerative potential than SB [20,31]. This fact can be demonstrated by the greater reduction of H_2_O_2_-induced oxidative damage in skin cells pretreated with DHS than those treated with SB [32]. However, the recently published phototoxic potential of QE and DHS stands in sharp contrast to the supposed antioxidant and skin treatment potential [33,34]. Therefore, the anti-inflammatory potential of newly recognized components, such as DHS needs to be investigated further.

In this study, DHS as the enantiomeric mixture of 2,3-dehydrosilybin A and 2,3-dehydrosilybin B was synthetized in bulk as described previously [35]. Briefly, silybinin was dissolved in glacial acetic acid and refluxed in the presence of iodine. A simple hydrolysis with hydrochloric acid in wet ethanol afforded the desired product. The study’s main focus lies in the analysis of the molecular mechanism for the effect of DHS on inflamed fibroblasts at the level of the inflammatory pathway, from their activation to the downstream production of pro-inflammatory mediators.

## 2. Results

### 2.1. Cytotoxicity Profiles of the Studied Component of Silymarin and Indomethacin

**Figure 1:**
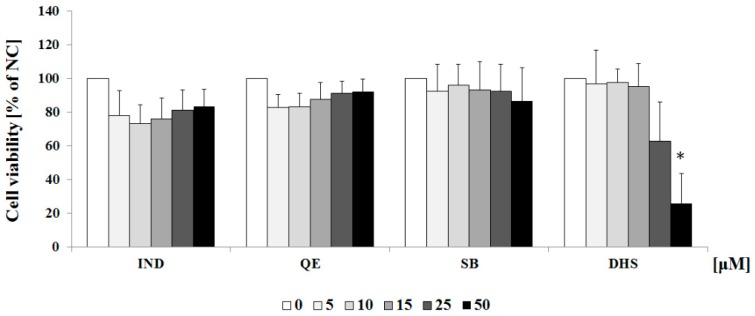
Cytotoxicity of QE, SB, DHS and IND on NHDF following direct 24 h exposure to flavonoids at different concentrations. Data are presented as the percentage cell viability relative to the control group NC (set to 100%) and represented as mean ± SEM (*n* = 3). Significant cytotoxicity was indicated as * *p* < 0.05 vs. control cells (0.1% vehicle DMSO in serum-free culture medium). NC: negative control (0.1% vehicle DMSO in serum-free culture medium).

First, we performed MTT experiments to explore the cytotoxicity of selected silymarin components, as well as the anti-inflammatory drug indomethacin IND. NHDF (normal human dermal fibroblasts) were exposed to each flavonoid (DHS, SB, QE) or IND at a concentration range from 0 to 50 μM for 24 h. The results showed that SB, QE and IND have no significant cytotoxic effect on NHDF. However, DHS displayed considerable cytotoxic effect on NHDF. As can be seen from the graph of Figure 1, 25 μM DHS slightly reduced the mitochondrial dehydrogenase activity and the significant reduction in the mitochondrial function was observed for 50 μM DHS. Based on the results, the concentrations < 15 μM of compounds were considered safe.

### 2.2. Effect of Subtoxic Concentrations of Selected Components of Silymarin on Cytokine and Chemokine Secretion

**Figure 2:**
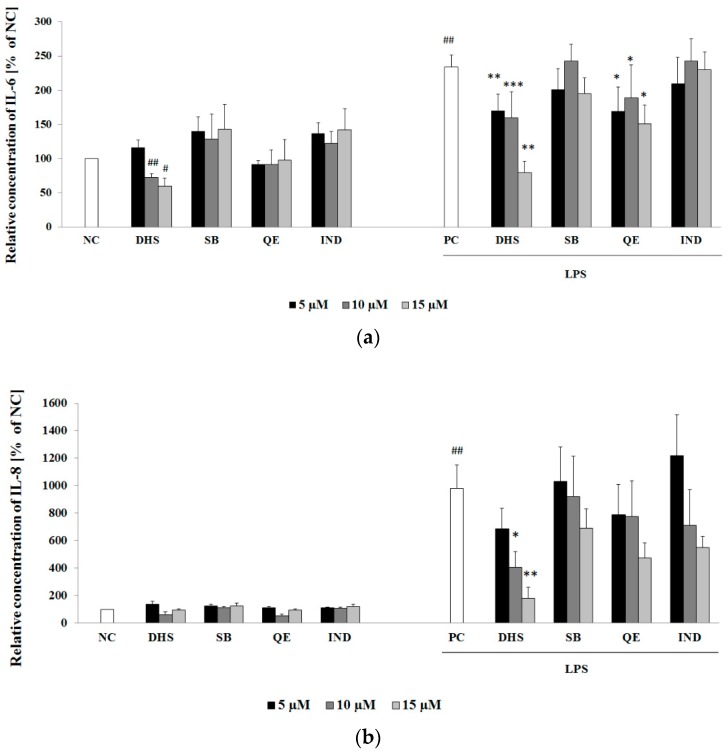
Cytokine IL-6 (**a**) and chemokine IL-8 (**b**) secretion into the medium were determined by ELISA assay, after 24 h treatment with 5, 10 and 15 μM QE, SB, DHS or IND with or without previous 6 h of LPS challenging. Data are presented as mean percentage relative to NC ± SEM (*n* = 3), significantly different from NC (^#^ *p* < 0.05; ^##^ *p* < 0.01) or PC (* *p* < 0.05; ** *p* < 0.01; *** *p* < 0.001). NC: negative control (0.1% vehicle DMSO in serum-free culture medium), PC: positive control (6 h of LPS stimulation followed by 24 h incubation with 0.1% vehicle DMSO in serum-free culture medium).

The high levels of pro-inflammatory cytokine IL-6 and chemokine IL-8 correlate with the severity of inflammation, and these are widely used as markers of inflammation [36,37]. The 6 h treatment with LPS increased the release of IL-6 and IL-8 into the culture medium (positive control PC; Figure 2a,b, respectively). At the given concentration of LPS, the stimulated NHDF displayed approximately 2-fold higher release of IL-6 compared to negative control NC (Figure 2a). As shown in Figure 2b, LPS was a more effective inducer of IL-8 release (10-fold of the negative control levels) than of pro-inflammatory cytokine IL-6 release. A subsequent 24 h exposure of LPS-challenged cells to DHS or QE resulted in a significant suppression of cytokine IL-6 production for each substance in the concentration range of 5–15 μM (Figure 2a). As for the inhibition of the IL-8 release, QE in contrast to DHS was shown to be ineffective. In addition, treatment with DHS demonstrated a dose-dependent reduction of the LPS-stimulated production of pro-inflammatory cytokines. The application of SB or the anti-inflammatory drug IND revealed no significant effects in the same concentration range.

### 2.3. Western Blotting Analysis of Molecular Targets of the Potential Anti-Inflammatory Action of Subtoxic Concentrations of Selected Components of Silymarin

**Figure 3:**
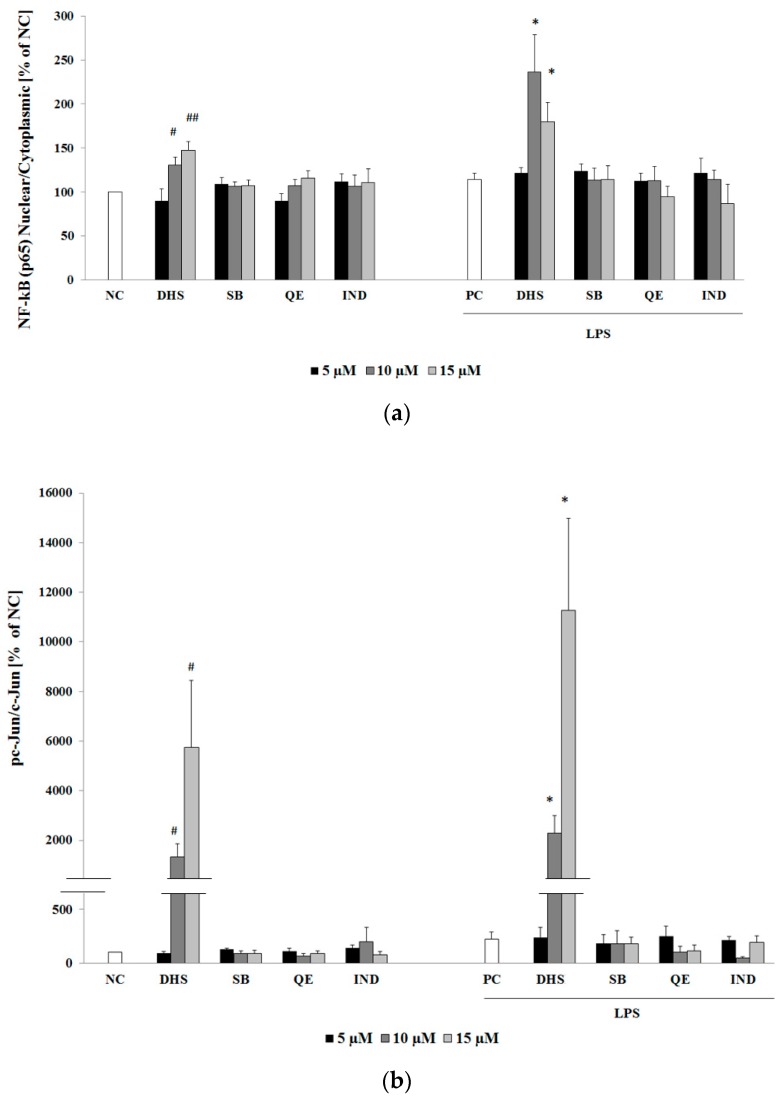

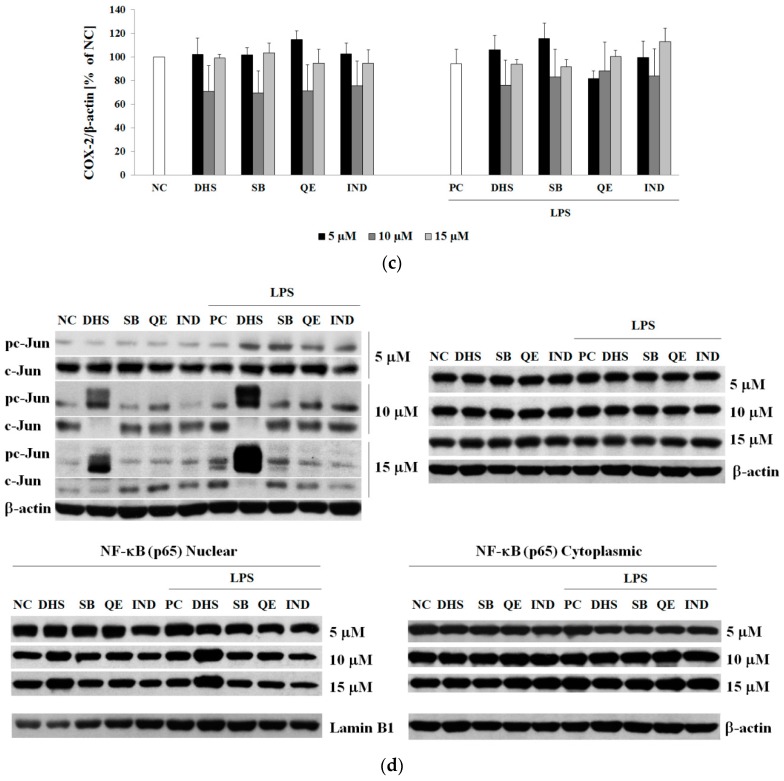
Evaluation of the selected silymarin components effect on transcription factor NF-κB (subunit p65) nuclear translocation, transcription factor AP-1 activation (phosphorylation of subunit c-Jun) and enzyme COX-2 protein levels with/without LPS pretreatment by densitometric analysis of Western blot bands. The analysis of transcription factors NF-κB p65 (**a**) nuclear translocation or AP-1 activation via c-Jun phosphorylation (**b**) or endogenous protein COX-2 expression (**c**) was performed on cytoplasmic/nuclear or whole-cell lysates of NHDF. Following 6 h of pre-incubation with or without LPS, NHDF were exposed to 5, 10 and 15 μM QE, SB, DHS or IND for a further 24 h. Beta-actin or Laminin B1 were used as a loading control, for whole-cell and cytoplasmic or nuclear lysates, respectively. Data are presented as a mean percentage relative to NC ± SEM (*n* = 5), significantly different from NC (^#^ *p* < 0.05; ^##^ *p* < 0.01) or PC (* *p* < 0.05). NC: negative control (0.1% vehicle DMSO in serum-free culture medium), PC: positive control (6 h of LPS stimulation followed by 24 h incubation with 0.1% vehicle DMSO in serum-free culture medium). (**d**) Representative Western blot.

NF-κB and AP-1 are transcription factors related to an inflammatory process. These factors are strongly implicated in an initiation of pro-inflammatory target gene expression, e.g., IL-6, IL-8 or COX-2 [13,38,39]. Therefore, we determined the protein levels of the aforementioned factors NF-κB (subunit p65) and AP-1 (subunit c-Jun) by Western blotting using the same experimental conditions (Figure 3a,b,d). Surprisingly, rather than suppressing it, 10 μM and 15 μM DHS increased the cytoplasmic-nuclear shuttling of the p65 component of NF-κB or phosphorylation of c-Jun the subunit of AP-1. The effect of DHS was more obvious under LPS-induced inflammatory conditions. Thus, LPS and DHS displayed synergy and/or cooperativity in a prolonged transcription factor NF-κB and AP-1 upregulation. Western blots also revealed much stronger dose-dependent AP-1 activation when compared to NF-κB. The phosphorylation of c-Jun results in the activation of the transcription factor AP-1 [40]. After 24 h exposure to DHS at the highest concentration used, NHDF with or without LPS-pretreatment showed approximately 120 to 150-fold or 6 to 9-fold increase in the phospho-c-Jun/c-Jun ratio, respectively, compared to the untreated cells (Figure 3b,d). With regard to the fact that LPS itself did not induce the cytoplasmic-nuclear shuttling of NF-κB or the phosphorylation of c-Jun within this prolonged experiment, we may hypothesize that DHS extended LPS-induced NF-κB nuclear translocation.

The traditional anti-inflammatory drug IND is a well-known non-selective inhibitor of the inducible pro-inflammatory enzyme COX-2, a downstream target of NF-κB signaling. Hence, we decided to evaluate the effect of DHS, SB and QE on the protein level of COX-2 in comparison to equally concentrated IND under the same experimental conditions as mentioned previously. In contrast to our expectations, neither the tested flavonoids, nor NSAID were able to regulate the level of COX-2 (Figure 3c). On the other hand, COX-2 levels in NHDF did not respond to 6 h of the LPS-challenge. This could be explained by the fact that once activated, the inducible enzyme COX-2 undergoes protein degradation at a relatively rapid rate [41].

### 2.4. Silymarin Component Effects on Cytokines IL-6 and IL-8 and Upstream Transcription Factors NF-κB (p65) or AP-1 (c-Jun) mRNA Levels

**Figure 4:**
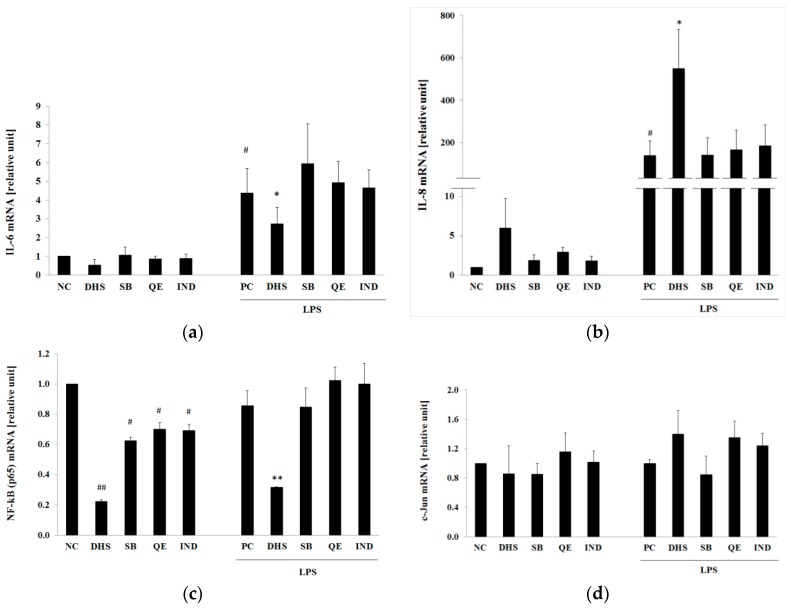
Effects of the selected silymarin components and IND on IL-6 (**a**); IL-8 (**b**); NF-κB (p65) (**c**) and AP-1 (c-Jun) (**d**) mRNA levels with or without the previous LPS challenge. The gene expression analysis was conducted using RT-PCR at 24 h after exposure to 10 μM DHS, SB, QE or IND with or without previous 6 h LPS challenge. RPL13A was used as an internal standard. Data are presented in arbitrary units as mean relative to NC ± SEM (*n* = 5), significantly different from NC (^#^ *p* < 0.05; ^##^ *p* < 0.01) or PC (* *p* < 0.05; ** *p* < 0.01). NC: negative control (0.1% vehicle DMSO in serum-free culture medium), PC: positive control (6 h of LPS stimulation followed by 24 h incubation with 0.1% vehicle DMSO in serum-free culture medium).

The evaluation of mRNA expression of cytokines and transcription factors in the treated NHDF was performed by real-time PCR (RT-PCR). LPS-treatment for 6 h significantly enhanced the expression levels of the pro-inflammatory IL-6 or chemotactic IL-8 cytokines (Figure 4a,b, respectively). To examine whether there is a correlation between the protein level of selected inflammatory markers measured by ELISA (Figure 2) and mRNA level, we used DHS at a concentration of 10 μM, which was shown to be effective in the reduction of both of these markers when compared to equal concentrations of SB, QE and IND. Treatment with the selected concentration of DHS was not accompanied by such an extensive increase of pro-inflammatory/pro-apoptotic transcriptional factor AP-1 activation observed at a higher concentration (15 μM) (see Figure 3b).

As shown in Figure 4a, the DHS suppressed the IL-6 mRNA expression induced by 6 h of LPS pretreatment. Thus, the IL-6 gene expression results supported the findings from the ELISA assay. However, DHS affected IL-8 mRNA level in the opposite direction to that expected. As shown in Figure 4b, DHS markedly increased IL-8 mRNA level in the cells pretreated with LPS (approx. 600–800 times higher than in the NC). Another discrepancy between the mRNA and protein expression was observed for the transcription factor NF-κB (p65) (Figure 4c), where DHS significantly reduced the NF-κB (p65) mRNA level increased by the LPS-pretreatment. However, the LPS challenge (PC) did not markedly stimulate the NF-κB (p65) gene expression when compared with the untreated cells (NC). Therefore, the suppressive effect of DHS seems to be independent of the previous LPS challenge. This hypothesis is supported by the fact that DHS also significantly decreased the NF-κB (p65) mRNA level to a similar degree also in LPS-untreated group (NC) (Figure 4c). Interestingly, other tested substances SB, QE and IND were also able to significantly reduce the basal mRNA level of NF-κB (p65) in LPS-untreated cells. However, these substances failed to exert the same effect in the LPS-treated cells. As can be seen from the Figure 4d, the AP-1 (c-Jun) mRNA levels were not significantly affected by any of the tested substances with or without the previous LPS-challenge. However, the lack of statistically significant difference between the means of treated and non-treated cells could be explained by a high variability in the individual responses of the fibroblasts derived from different donor cell sources.

### 2.5. Western Blotting Analysis of Cotreatment of Nontoxic Concentrations of Dehydrosilybin and Lipopolysaccharide

**Figure 5:**
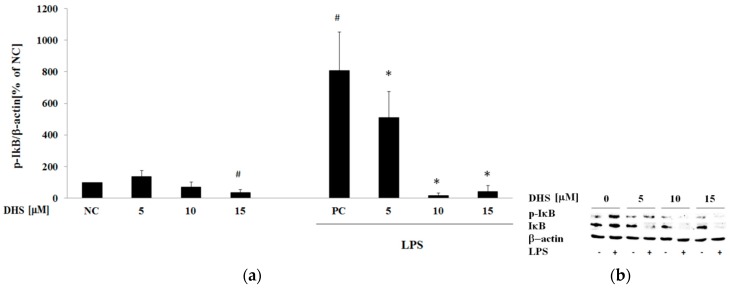
Western blot analysis of NF-κB activation. The evaluation of transcription factor NF-κB activation by determination of the phosphorylated/non-phosphorylated form of NF-κB inhibitor IκB (subunit IκB-α) after the 6 h exposure period to 5, 10 and 15 μM DHS with or without LPS-cotreatment. Beta-actin served as a loading control. (**a**) Graphical presentation of the results, where IκB phosphorylation is expressed as the mean percentage relative to NC ± SEM (*n* = 3), significantly different from NC (^#^ *p* < 0.05) or PC (* *p* < 0.05). NC: negative control (0.1% vehicle DMSO in serum-free culture medium), PC: positive control (6 h of LPS stimulation with 0.1% vehicle DMSO in serum-free culture medium). Quantification was performed by densitometric analysis of Western blot bands. (**b**) Representative blot images of 3 replicates.

Next, we examined the possible antagonistic or cooperative activity of LPS and selected the most effective component of silymarin according to results obtained from the aforementioned post-treatment protocol. In resting cells, the DNA-binding activity of NF-κB is inhibited by the cytoplasmic association with the endogenous NF-κB inhibitor IκB. Inflammatory/stress conditions promote phosphorylation of IκB by IκB kinase (IKK) resulting in its ubiquitination and degradation, enabling NF-κB nuclear translocation [4]. We performed Western blotting of phospho-IκB-α and IκB-α after 6 h treatment of NHDF with 5, 10 and 15 μM DHS with or without LPS-cotreatment. However, as can be seen from the results of phospho-IκB-α band intensity normalized to β-actin (Figure 5a), 10 and 15 μM DHS significantly decreased the LPS-induced phosphorylation of IκB-α, revealing the opposite of the expected effect, when compared to previous results.

At the same time, DHS and LPS coincubation reduced the total IκB-α protein level in treated cells (Figure 5b). Taken together, we concluded that 10 and 15 μM DHS inhibited IκB-α protein expression, thus making the restoration of the inhibitor levels following its LPS-induced phosphorylation/degradation impossible.

### 2.6. DHS Effect on IκB mRNA Level with or without LPS Pretreatment

**Figure 6:**
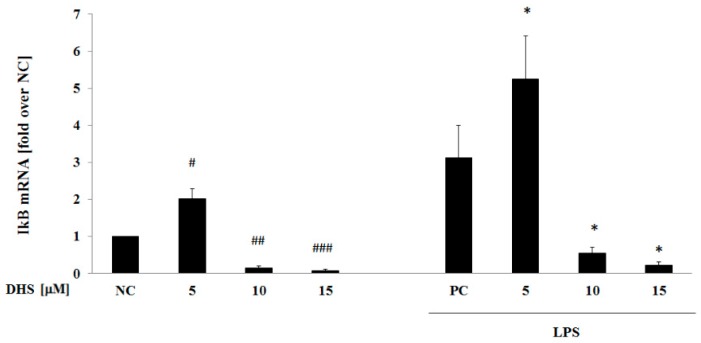
Effect of DHS on IκB mRNA level with or without previous LPS challenge. Gene expression analysis was conducted using quantitative RT-PCR after 24 h of exposure to 5–15 μM DHS with or without previous 6 h LPS challenge. RPL13A was used as an internal standard. Data are presented as the mean percentage relative to NC ± SEM (*n* = 3), significantly different from NC (^#^ *p* < 0.05; ^##^ *p* < 0.01; ^###^ *p* < 0.001) or PC (* *p* < 0.05). NC: negative control (0.1% vehicle DMSO in serum-free culture medium), PC: positive control (6 h of LPS stimulation followed by 24 h incubation with 0.1% vehicle DMSO in serum-free culture medium).

The inhibition of the IκB protein expression by 6 h exposure to DHS under inflammatory conditions induced by LPS (Figure 5) led us to question whether prolonged DHS exposure also reduces IκB gene expression when applied as a posttreatment. Similar effects to those observed in Western blot analysis, when LPS and DHS were applied as a 6 h co-treatment (Figure 5), were seen for 24 h NHDF post-treatment with 10 and 15 μM DHS (Figure 6). Interestingly, exposing NHDF to 5 μM DHS strengthened the effect of LPS-pretreatment resulting in an approximately 2-fold enhancement of IκB gene expression. These concentration-dependent effects of DHS exhibited the same trend regardless of LPS pretreatment. LPS-pretreatment in turn increased IκB mRNA levels (Figure 6).

### 2.7. DHS Effect on NF-κB DNA-Binding Activity with or without Cotreatment with Lipopolysaccharide

**Figure 7:**
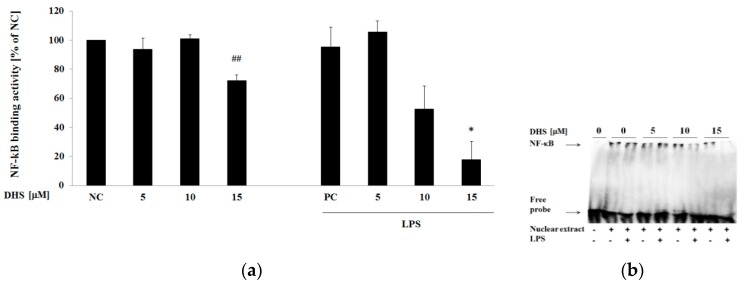
Effect of DHS on NF-κB binding activity performed by EMSA using nuclear extracts of NHDF treated with 5–15 μM DHS for 6 h or in the presence of LPS. (**a**) Graphical presentation of the results, where NF-κB binding activity is expressed as mean percentage relative to NC ± SEM (*n* = 3), significantly different from NC (**^##^** *p* < 0.01) or PC (* *p* < 0.05). NC: negative control (0.1% vehicle DMSO in serum-free culture medium), PC: positive control (6 h of LPS stimulation with 0.1% vehicle DMSO in serum-free culture medium). Quantification was performed by densitometric analysis of bands. (**b**) Representative gel image.

To uncover the whole picture of DHS’s effect on NF-κB, as the controversy between DHS’s effects on NF-κB protein level, gene expression and activation exists, and considering the discrepancy in IL-8 gene and protein expression downstream of NF-κB, we decided to perform an evaluation of NF-κB binding to DNA target sequences. When the protein binds to the probe corresponding to the NF-κB p65 DNA-binding motif, the electrophoretic mobility is reduced (gel retardation assay) compared to the free probe. On the basis of the IκB results, we hypothesized that the enhanced activation of NF-κB would result in augmented binding activity of NF-κB to the promoter region. However, Figure 7 shows the opposite result after 6 h exposure to DHS. This treatment reduced the binding of NF-κB to the corresponding DNA sequence significantly at the highest concentration tested (15 µM) and in a dose-dependent manner in the presence of LPS. Moreover, this effect was more pronounced when NHDF was exposed to LPS at the same time. DHS, at a concentration of 15 μM decreased the NF-κB binding activity to approximately 70% of the basal level (Figure 7a) and to approximately 30% of the binding activity induced by LPS. On the other hand, under our conditions, NHDF showed quite a high basal NF-κB binding activity and the LPS alone did not induce any significant changes in DNA-binding activity of NF-κB.

## 3. Discussion

Inflammation is not primarily a pathological process. This physiologic action is, first of all, a defense mechanism preventing the spread of tissue infection, since wounds are often contaminated with pathogens. Inflammation becomes detrimental only if it is not properly terminated and passes into a chronic state indicated by a persistent production of pro-inflammatory mediators [3]. However, the dividing line between normal and excessive inflammatory response is not easy to determine, especially using in vitro analysis. Thus, in our study, in order to distinguish between normal and excessive inflammatory response, we used a wound scratch assay to mimic a normal healing response and an additional LPS treatment simulating the later. 

In our study, the cytotoxicity testing of selected silymarin components in normal human dermal fibroblasts revealed that only DHS had the capacity to decrease cell viability significantly at the highest concentration tested (50 µM). Similar results were reported by Svobodova et al. in HaCaT keratinocytes and mouse fibroblasts [32]. According to the authors, this effect could be associated with DHS’s higher lipophilicity in comparison to that of SB allowing passage across the membrane [32,42] and interaction with membrane lipids and proteins [42,43]. As reported by Pyszkova et al., the increased cytotoxicity of DHS probably comes from the fact that DHS is more reactive when compared to the other silymarin components. The presence of the C-2,3 double bond connected with a C-3 hydroxyl group not only makes DHS a more potent free radical scavenger, but also increases its cytotoxic potential [44].

Pretreatment with silymarin was shown to attenuate the LPS-induced COX-2 mRNA expression in fibroblasts [33]. In contrast, we observed no significant effect of posttreatment with any of the studied constituents on the modulation of this inducible isoform of pro-inflammatory enzyme using western blot analysis. However, considering the fact that COX-2 was not significantly upregulated by LPS, its downregulation could be detrimental to a normal healing response in wound scratch assay.

The results of the present study show that DHS diminished the LPS-induced release of pro-inflammatory cytokine IL-6 and chemokine IL-8 into the surroundings in a dose-dependent manner and to a higher extent than the other components of silymarin that were tested. On the other hand, DHS also slightly decreased the level of IL-6 in cells not stimulated with LPS, and thus may counteract the physiological proliferative capacity of IL-6 in skin cells essential for wound healing [45,46]. The mRNA analysis supported DHS’s ability to reduce cytokine IL-6 induction by LPS. However, a contrasting result was obtained for the chemoattractant IL-8, which showed that DHS posttreatment of LPS-challenged cells resulted in a significant increase in the level of IL-8 mRNA. Besides polymorphonuclear neutrophil recruitment leading to the subsequent elimination of bacteria from the wound, IL-8 has been found to locally contribute to neoangiogenesis, fibroblasts proliferation and epidermal reepithelialization. On the other hand, the excessive production of IL-8 may result in fibroplasia, the characteristic feature of psoriasis [47]. The discrepancy between the IL-8 mRNA and ELISA results can be explained by the fact that a time delay exists between peaks in mRNA and subsequent protein synthesis. Another possible hypothesis could be a DHS-induced posttranslational modification or inhibition of protein secretion. The IL-8 mRNA is stabilized by the MAPK/p38 pathway [48], however, MAPK/p38 activation remained mostly unaffected by DHS treatment (see Appendix A).

At the molecular level, once the infection is eliminated, NF-κB activity should drop to its basal level [41]. On the other hand, pathological inflammatory processes are primarily associated with an incorrect termination of NF-κB signaling. The surface components of gram-negative bacteria, mainly LPS, are able to directly activate NF-κB and AP-1 signaling pathways and thus, increase production of cytokines IL-6 or IL-8 in dermal cells. The resident skin cells, such as fibroblasts, act as nonprofessional immune cells. They may induce and intensify the initial pro-inflammatory signal for the activation and directed influx of circulating neutrophils towards the infected area [49]. In our study, we observed a dose-dependent DHS-induced reduction of LPS-promoted NF-κB (p65) activation. This was based on the well-known fact, that phosphorylation and degradation of NF-κB inhibitor IκB allows NF-κB to enter into the nucleus and exert its transcriptional activity. However, the simultaneous Western blotting evaluation of total IκB protein expression revealed a DHS-induced reduction in the total protein level when LPS was added concurrently. Thus, it is likely that DHS prevented *de novo* synthesis of IκB protein following its LPS-dependent degradation. In a post-treatment experimental setup, NF-κB (p65) nucleocytoplasmic shuttling and the decreased *IκB* gene expression in LPS-challenged cells were enhanced to a greater extent following prolonged treatment with DHS than following short-term treatment. Because NF-κB is able to terminate its own transcriptional activity via *de novo* synthesis of its own inhibitor IκB, which can disengage NF-κB from DNA [41,50], we interpreted the results obtained by Western blotting as a reinforcement of the NF-κB activation by treatment with DHS under inflammatory conditions induced by LPS. 

Unexpectedly, despite DHS promoting the translocation of NF-κB into the nucleus, we observed diminished a DNA-binding in fibroblasts treated concurrently with DHS and LPS for 6 h in a dose-dependent manner. This effect could be beneficial in the regulation of an excessive inflammatory response. However, as can be seen from our observations, LPS did not markedly increase NF-κB DNA-binding activity under our experimental conditions. Therefore, it seems likely, that DHS prevents a normal physiological response to infection. In addition, 6 h treatment with DHS in the absence of LPS resulted in the significant inhibition of NF-κB DNA-binding at the highest (15 μM) concentration used. Besides its pro-inflammatory capacity, NF-κB controls cell proliferation and differentiation as well as protecting cells from apoptosis by induction of anti-apoptotic genes [2,51]. The capacity of DHS to inhibit the constitutive DNA-binding activity of the transcription factors NF-κB and AP-1, in terms of the growth prevention of cancer cells, has been reported to be higher than that of SB [52]. However, this effect may be related to the DHS-mediated inhibition of NF-κB activity, as it was shown that NF-κB-incompetent cells may be more sensitive to TNF-α induced apoptosis [53]. The expression of anti-apoptotic genes induced by NF-κB may protect cells from TNF-α-induced apoptosis, which may contribute to inflammation [51]. On the other hand, controlled apoptosis may prevent exaggerated inflammation, hence allowing the elimination of infected and damaged cells [2,54]. Thus, NF-κB exerts both pro and anti-inflammatory functions.

During inflammation, the induction of chemokine IL-8 expression requires the cooperative transcriptional activity of both NF-κB and AP-1 factors [39]. According to our results, DHS dose-dependently induced AP-1 activation after prolonged exposure. This effect was much more prominent in LPS-pretreated cells. Thus, the transcription factors collaboration could be associated with observed increased level of IL-8 mRNA. Besides its pro-inflammatory action, the prolonged activation of c-Jun (the transcription factor AP-1 subunit) leads to apoptosis [55]. On the other hand, considering the incubation period, the involvement of DHS metabolites cannot be excluded, which would provide a subject for further study.

## 4. Materials and Methods

### 4.1. Chemicals

Dulbecco’s modified Eagle’s medium (DMEM), Ham-F12 Nutrient Mixture, heat-inactivated fetal bovine serum (FBS), stabilized penicillin-streptomycin solution (P/S), amphotericin B, hydrocortisone, adenine, insulin, epidermal growth factor, 3,3′,5-triiod-l-thyronin, trypsin-EDTA (0.25%), ampicillin, amino acids (l-histidine, l-isoleucine, l-methionine, l-tryptophan, l-tyrosine), 3-(4,5-dimethylthiazol-2-yl)-2,5-diphenyltetrazolium bromide (MTT), DMSO and LPS from Pseudomonas aeruginosa were purchased from Sigma-Aldrich (Prague, Czech Republic). Polyclonal antibodies goat anti-β-actin (sc-1616), rabbit anti-c-Jun (sc-1694), rabbit anti-NF-κB p65 (sc-372) and goat anti-COX-2 (sc-1747), monoclonal antibodies mouse anti-lamin B1 (sc-377000) and mouse anti-phospho-c-Jun (sc-822) as well as horseradish peroxidase (HRP)-conjugated secondary antibodies goat anti-rabbit (sc-2004), rabbit anti-goat (sc-2922) and rabbit anti-mouse (sc-2005) were obtained from Santa Cruz Biotechnology (Santa Cruz, CA, USA). The polyclonal antibodies rabbit anti-IκBα (#9242) and rabbit anti-phospho-IκBα (#9241) were supplied by Cell Signaling Technology (Beverly, MA, USA).

### 4.2. Tested Compounds

SB and DHS were provided by the Institute of Microbiology of the Czech Academy of Science, Laboratory of Biotransformation, Prague, Czech Republic. The isolation of SB from the crude extract of the seeds of milk thistle (silymarin) as well as its following oxidation, that gives rise the derivative DHS, has been described in detail previously [56,57]. QE and IND were purchased from Sigma-Aldrich (Czech Republic). Millimolar concentrations of stock solutions of compounds were diluted in dimethyl sulfoxide (DMSO). Experimental solutions in serum-free medium contained 0.1 % (*v*/*v*) final concentration of DMSO.

### 4.3. Cell Culture

Primary normal human dermal fibroblasts (NHDF) were isolated with informed consent from the skin specimens of healthy volunteers. The study was approved by the local Ethical Committee (University Hospital Olomouc; Ref. No. 41/09) and carried out in accordance with the Code of Ethics of the World Medical Association. The skin sections were obtained during plastic surgery, as previously reported [58]. The cell origin and morphology of human fibroblasts was verified by the Histology Department of the University Hospital Olomouc.

Briefly, NHDF were cultured in DMEM medium supplemented with 10% (*v*/*v*) FBS and 1% (*v*/*v*) P/S and maintained under standard culture conditions (37 °C, 5% CO_2_, 95% relative humidity).

### 4.4. Cell Viability Assay

NHDF were seeded into the wells of the 96-well plates at a density of 0.8 × 10^3^ cells/well. The following day, solutions of tested compounds (DHS, SB, QE, IND) within a concentration range 5–50 μM or negative control (0.1% (*v*/*v*) DMSO) and positive control (1.5% (*v*/*v*) Triton X-100) in serum-free DMEM medium containing P/S were applied for 24 h. Cell viability was evaluated using a standard MTT assay, based on the ability of mitochondrial enzymes of living cells to metabolize yellow water-soluble tetrazolium salt into insoluble purple formazan. Formazan crystals were dissolved in ammonia-containing dimethyl sulfoxide and spectrophotometrically quantified at 540 nm.

### 4.5. Treatment Protocols

For experiments, the third passage of cell culture was seeded in Petri dishes (10 cm diameter) at a cell density of 0.5 × 10^5^ cells/cm^2^ and cultivated until reaching confluence. The following day, a scratch was made across the monolayers using a 10 mL pipette tip to mimic a presence of a wound. After that, the cells were washed with phosphate-buffered saline (PBS) to remove detached cells.

Wound inflammation was induced by preincubation with LPS (final concentration 10 μg mL^−1^) for 6 h. We used LPS from gram-negative bacteria P. aeruginosa that is a known inducer of inflammation [49]. The dose of bacterial LPS (10 μg mL^−1^) was selected on the basis of previously performed experiments in our laboratory, which has also been frequently described elsewhere [49,59]. Following the incubation period, the cell monolayers were washed with PBS prior to the addition of solutions of tested compounds (DHS, SB, QE, IND) at concentrations of 5 μM, 10 μM or 15 μM in serum-free DMEM medium containing P/S. After 24 h of incubation time, the medium and cells were harvested for analysis. A negative control (scratched cells growing in corresponding serum-free DMEM medium containing vehicle 0.1% (*v*/*v*) DMSO without any additional treatment) and a positive control (6h-LPS-treated scratched cells prior to incubation in serum-free DMEM medium) were analyzed as well. Experiments were performed independently using cells obtained from three to five different donors.

To investigate the possible antagonistic or cooperative effect of the most effective component of silymarin with LPS, the coincubation experiments have been performed as well. Thus, in the next part of our investigation, the co-treatment protocol was performed. After 6 h of simultaneous treatment with LPS (10 μg mL^−1^) and 5 μM, 10 μM or 15 μM concentrations of selected component, the cells were harvested and analyzed. At the same time, the negative control (the scratched cells growing in serum-free DMEM medium containing 0.1% (*v*/*v*) DMSO without any additional treatment) and the positive control (6 h of LPS-treatment of scratched cells in serum-free DMEM medium) were analyzed as well. Experiments were performed independently using cells obtained from three different donors.

### 4.6. Enzyme-Linked Immunosorbent Assay (ELISA)

Human IL-6 and IL-8 levels in cell culture supernatants were measured with ELISA Development Kits from PeproTech (Prague, Czech Republic) according to the manufacturer’s protocol.

### 4.7. Western Blot Analysis

After the treatment, cell monolayers were washed with cold (4 °C) PBS, scraped and collected by centrifugation at 4700 rpm for 5 min at 4 °C. The resulting pellet was subjected to lysis. Whole cell lysates were prepared using a RIPA buffer (for the preparation, see Reference [20]). The separation of cytosolic and nuclear extracts was performed as described previously [32]. Protein contents were quantified by the Bradford assay. Equal amounts of the extracted samples (20 μg of protein) were loaded onto 10% SDS-polyacrylamide gel and subjected to electrophoresis. The separated proteins were transferred onto polyvinylidene difluoride (PVDF) membrane (ImmobilonP, Millipore, Billerica, MA, USA). Subsequently the membranes were blocked with 5% nonfat milk in tris-buffered saline (TBS) with 0.1% Tween 20 for 2 h, then probed with the relevant primary antibodies and HRP-conjugated secondary antibodies. The protein visualization was carried out using chemiluminescent photographic detection. The relative band intensities were quantified by densitometric analysis using ImageJ software (1.48v, National Institutes of Health, Bethesda, MD, USA).

### 4.8. RNA Isolation and Quantitative Real-Time RT-PCR

Immediately after completing the experiment, total RNA was extracted using Nucleospin^®^ RNA kit (MachereyNagel, Bethlehem, PA, USA) in accordance with the manufacturer’s instructions. DNAseI treated RNA samples (1 μg) were reverse transcribed into cDNA using the M-MLV RT kit (Invitrogen, Waltham, MA, USA) as previously described [60]. To achieve the amplification of generated cDNA, we performed real-time PCR in a StepOnePlus^TM^ Real-Time PCR System (Applied Biosystems, Foster City, CA, USA) with specific primers designed with Primer Express Software (Version 3.0.1, Applied Biosystems). The primer sequences (5′→3′) used for real-time RT-PCR amplification were: for target gene RPL13A, GAGGTATGCTGCCCCACAAA Primer Express Software (sense), GTGGGATGCCGTCAAACAC (antisense); for IL-6, CACACAGACAGCCACTCACC (sense), TTTCACCAGGCAAGTCTCCT (antisense); for IL-8, GTGCAGTTTTGCCAAGGAGT (sense), CTCTGCACCCAGTTTTCCTT (antisense); for NF-κB (p65), TAGGAAAGGACTGCCGGGAT (sense), CCGCTTCTTCACACACTGGA (antisense); for IκB, CCATGGTCAGTGCCTTTTCT (sense), GTCAAGGAGCTGCAGGAGAT (antisense); for AP-1 (c-Jun) GAAGTGTCCGAGAACTAAAG (sense), AAAAGTCCAACGTTCCGTTC(antisense). The real-time assays were carried out under following amplification conditions: initiation at 95 °C for 10 min, followed by 40 cycles of denaturation at 95 °C for 15 s and annealing and extension 60 °C for 60 s. Relative mRNA levels were calculated using the comparative C_T_ method (2^−∆∆CT^) and normalized to housekeeping gene RPL13A expression.

### 4.9. Electrophoretic Mobility Shift Assay (EMSA)

DNA-binding activity of transcription factor NF-κB p65 was estimated in cell nuclear lysates which were prepared as mentioned above. The oligonucleotide sequence containing NF-κB DNA-binding motif used for EMSA (5′-AGTTGAGGGGACTTTCCCAGGC-3′) was provided by Eurogentec (Seraing, Belgium). It was annealed with its reverse complement and used as a double stand. The EMSA was performed under non-denaturating conditions as described previously [61]. Following the electrophoretic separation, the proteins were electrically transferred to PVDF membrane, then probed with a streptavidin-HRP conjugate and visualized by the enhanced chemiluminescence substrate PlusECL (PerkinElmer, Boston, MA, USA). The chemiluminescent signal was revealed using ChemiDoc^TM^ XRS+ imaging system (BioRad, Hercules, CA, USA).

### 4.10. Statistical Analysis

Unless stated otherwise, the measurements were carried out in triplicates and data of at least three independent experiments (*n* = 3) with different cell donors were expressed as the mean ± SEM. Comparison between experimental groups was made by the Student’s t-test and *p* value bellow 0.05 (*), 0.01 (**), or 0.001 (***) were determined to be statistically significant.

## 5. Conclusions

Taken together, we conclude that DHS has a strong potential in the deregulation of physiological inflammatory responses. We realized that DHS decrease the IL-6 and IL-8 production but induced the significant upregulation of IL-8 mRNA associated with NF-κB and AP-1 activation. In this way, DHS may compromise wound-healing machinery, when compared to other tested silymarin components or indomethacin. Therefore, the long-term topical application of this powerful component should be revised, especially for the treatment of skin infections or chronic inflammatory skin diseases.

## Figures and Tables

**Figure A1:**
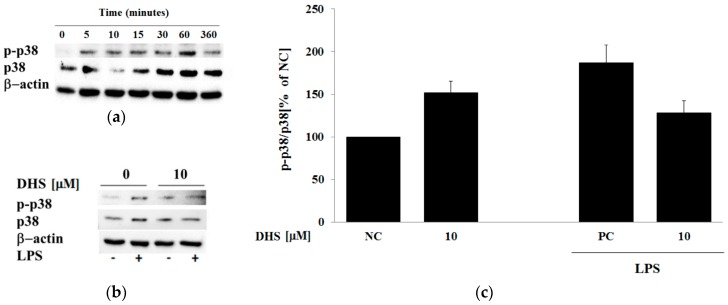
Western blot analysis of MAPK/p38 activation stimulated by LPS. (**a**) Time course measurement; NHDF were stimulated or unstimulated with 10 μg mL^−1^ of LPS for 5, 10, 15, 30, 60 or 360 min. Beta-actin served as a loading control. Representative blot image. (**b**) The evaluation of the MAPK/p38 activation using the phospho/total protein ratio after the exposure to 10 μM DHS for 10 min with or without LPS-cotreatment. Beta-actin served as a loading control. Representative blot image. (**c**) Schematic representation of p38 phosphorylation after the exposure to 10 μM DHS for 10 min with or without LPS-cotreatment. Results are expressed as mean percentage relative to NC ± SEM (*n* = 2). NC: negative control (0.1% vehicle DMSO in serum-free culture medium), PC: positive control (10 min of LPS stimulation with 0.1% vehicle DMSO in serum-free culture medium). Quantification was performed by densitometric analysis of Western blot bands.
